# Effects of laterally wedged insoles on symptoms and disease progression in medial knee osteoarthritis: a protocol for a randomised, double-blind, placebo controlled trial

**DOI:** 10.1186/1471-2474-8-96

**Published:** 2007-09-24

**Authors:** Kim Bennell, Kelly-Ann Bowles, Craig Payne, Flavia Cicuttini, Richard Osborne, Anthony Harris, Rana Hinman

**Affiliations:** 1 Centre for Health, Exercise & Sports Medicine, School of Physiotherapy, University of Melbourne, Australia; 2 Department of Podiatry, School of Human Biosciences, LaTrobe University, Melbourne, Australia; 3 Department of Epidemiology and Preventive Medicine, Alfred Hospital, Monash University, Melbourne, Australia; 4 AFV Centre for Rheumatic Diseases, Department of Medicine (RMH/WH), University of Melbourne, Australia; 5 Health Economics Unit, Monash University, Melbourne, Australia

## Abstract

**Background:**

Whilst laterally wedged insoles, worn inside the shoes, are advocated as a simple, inexpensive, non-toxic self-administered intervention for knee osteoarthritis (OA), there is currently limited evidence to support their use. The aim of this randomised, double-blind controlled trial is to determine whether laterally wedges insoles lead to greater improvements in knee pain, physical function and health-related quality of life, and slower structural disease progression as well as being more cost-effective, than control flat insoles in people with medial knee OA.

**Methods/Design:**

Two hundred participants with painful radiographic medial knee OA and varus malalignment will be recruited from the community and randomly allocated to lateral wedge or control insole groups using concealed allocation. Participants will be blinded as to which insole is considered therapeutic. Blinded follow up assessment will be conducted at 12 months after randomisation. The outcome measures are valid and reliable measures recommended for OA clinical trials. Questionnaires will assess changes in pain, physical function and health-related quality-of-life. Magnetic resonance imaging will measure changes in tibial cartilage volume. To evaluate cost-effectiveness, participants will record the use of all health-related treatments in a log-book returned to the assessor on a monthly basis. To test the effect of the intervention using an intention-to-treat analysis, linear regression modelling will be applied adjusting for baseline outcome values and other demographic characteristics.

**Discussion:**

Results from this trial will contribute to the evidence regarding the effectiveness of laterally wedged insoles for the management of medial knee OA.

**Trial registration:**

ACTR12605000503628; NCT00415259.

## Background

Osteoarthritis (OA) is a chronic, localised joint disease affecting approximately one-third of adults, with the disease prevalence increasing with advancing age [1]. The economic impact of knee OA is also a large and growing problem for health care systems. In Australia, the estimated financial costs of OA and other arthritic diseases in 2000 totalled almost 9 billion dollars [2]. Demographic predictions indicate that people aged over 65 years will comprise more than 20% of the population by 2040 [3], thus knee OA will only become more prevalent.

The knee is the most common lower limb site for OA, with the disease affecting the tibiofemoral and patellofemoral joints either in isolation or combination. The medial tibiofemoral compartment is the most commonly affected (medial 67% versus lateral 16% [4]). Patients with knee OA frequently report symptoms of knee pain and difficulty with activities of daily living, such as walking, stair-climbing and housekeeping [5]. Ultimately, pain and disability associated with the disease lead to a loss of functional independence and a profound reduction in quality-of-life.

Management strategies for knee OA may be regarded as *primary prevention *(reduction of risk factors to reduce disease incidence); *secondary prevention *(interventions to slow/prevent progression to serious disease) or; *tertiary prevention *(treatment of pain and disability) [6]. To date, most knee OA research has focussed on tertiary management strategies, primarily drug therapies. Although effective, drug therapies have side effects and are expensive [7]. Accordingly, recent knee OA clinical guidelines reinforce the importance of non-pharmacological strategies in the management of the condition [8,9] yet there is an absence of high quality evidence to support the use of such therapies [8]. Thus there is a clear need for future clinical trials to evaluate specific non-pharmacological therapies in order to better guide clinical decision-making.

Given that there is currently no cure for knee OA and the only established treatment for end-stage OA is costly joint replacement, slowing of structural disease progression is essential to help reduce the personal and societal burden of knee OA. Traditionally, disease progression has been assessed by measuring loss of joint space over time from serial x-rays. There is now increasing use of magnetic resonance imaging to measure knee cartilage volume as it has proved to be a valid and reproducible technique that is more sensitive to change than x-rays [10]. Given the absence of a sufficiently sensitive measure of structural change until recently, this has meant that few interventions have been tested as to their effect on disease progression.

Increased load across the joint is important in the pathogenesis of knee OA. Interventions that alter knee load may reduce symptoms and slow disease progression in patients with knee OA. Direct measurement of knee joint loads is not feasible because of the invasive nature of this in vivo method. However, gait analysis can calculate external joint-loading moments that are directly related to internal joint loads. The external knee adduction moment determines load distribution across the medial and lateral tibial plateaus, with force across the medial compartment almost 2.5 times that of the lateral [11]. This may explain the much higher prevalence of medial compared with lateral tibiofemoral joint OA.

The magnitude of the adduction moment is partly determined by the mechanical alignment of the knee. In medial knee OA, mechanical alignment becomes varus as the medial joint space narrows. Varus malalignment causes the ground reaction force vector to pass more medially to the knee joint centre, resulting in a higher knee adduction moment. Cross-sectional studies demonstrate that patients with knee OA have a higher knee adduction moment during walking when compared to healthy age-matched controls [12,13].

Recent research has found that a higher adduction moment is associated with more severe knee pain [14] and greater radiographic disease severity [15]. Severity of knee malalignment is also significantly associated with knee pain severity and physical function [16]. Longitudinal studies have demonstrated that as little as a one-unit increase in the adduction moment is associated with up to a 6.5-fold increase in the risk of disease progression [14,17]. Similarly, knee joint varus malalignment is also correlated with disease progression [16,18,19]. Given the importance of the knee adduction moment and joint alignment with regard to both symptom severity and disease progression in knee OA, conservative strategies to alter these biomechanical factors constitute a logical rehabilitative approach.

In 1987, Sasaki and Yasuda [20,21] first reported the potential of a laterally wedged insole in the shoe to treat medial knee OA. They demonstrated that the insole statically aligned the knee in a more upright position by shifting the calcaneus into a valgus position relative to the tibia. The authors concluded that such an alteration helped reduce excessive loading of the medial joint surface, leading to mitigation of knee pain. Biomechanical studies have since evaluated the effects of laterally wedged insoles on knee alignment and medial compartment loading. Ogata et al [22] demonstrated that lateral thrust of the knee (which forces the knee into varus alignment) was reduced with lateral wedges in people with normal and osteoarthritic knees. Similar results have been reported by more recent studies [23-25], with Kerrigan et al [24] demonstrating that a 5° laterally wedged insole significantly reduces the knee adduction moment by 6% in medial knee OA. Finally, Giffin et al [26] observed a significant varus to valgus shift in knee alignment on static radiographs with a lateral heel wedge although this has not been a consistent finding [27]. Thus there is evidence that lateral wedges can reduce varus malalignment and the adduction moment, two key biomechanical features that are associated with knee OA symptoms and disease progression.

However, despite their biomechanical effects, few randomised controlled trials have evaluated their clinical efficacy [28-30]. In 156 patients with medial knee OA, no significant effect of laterally wedged insoles on symptoms over 2 years was demonstrated, as compared to control insoles [28]. However, a significant reduction in non-steroidal anti-inflammatory drug intake and greater compliance was observed with laterally wedged insoles, leading the authors to conclude that the results favoured a beneficial effect of the intervention. A recent double-blind, randomized, crossover trial in 90 patients found no effect of 6 weeks of lateral wedge use on pain or [30]. The samples selected for these studies may partly explain the non-significant effect of laterally wedged insoles. Whilst patients demonstrated medial knee OA, selection criteria did not consider knee joint alignment. Significant effects are more likely in participants with evidence of varus malalignment, rather than in a diverse cohort where alignment is likely to range from valgus (in which lateral wedges could be detrimental) to neutral to varus.

As previously stated, it is also important to evaluate the effect of treatment on disease progression. Only one clinical trial has investigated the effect of lateral wedges on knee OA progression [29]. Using annual x-rays for two years, this study failed to show that lateral wedges slowed joint space narrowing over time compared to control insoles. This non-significant finding may relate to the use of relatively insensitive x-rays as a measure of disease progression, as well as to an inappropriate patient group and the use of a short rather than full length wedge. Given the paucity of data attesting to the structural effects of lateral wedges over time, further research is necessary using more sensitive methods of measuring disease progression such as magnetic resonance imaging. Furthermore, no study to date has evaluated the cost-effectiveness of lateral wedges.

The aim of this trial is to determine whether wearing lateral wedge insoles for 12 months improves pain, physical function and health-related quality of life, slows structural disease progression and is more cost-effective than control flat insoles in individuals with medial knee joint OA and varus malalignment.

## Methods/Design

### Design

**Figure 1:**
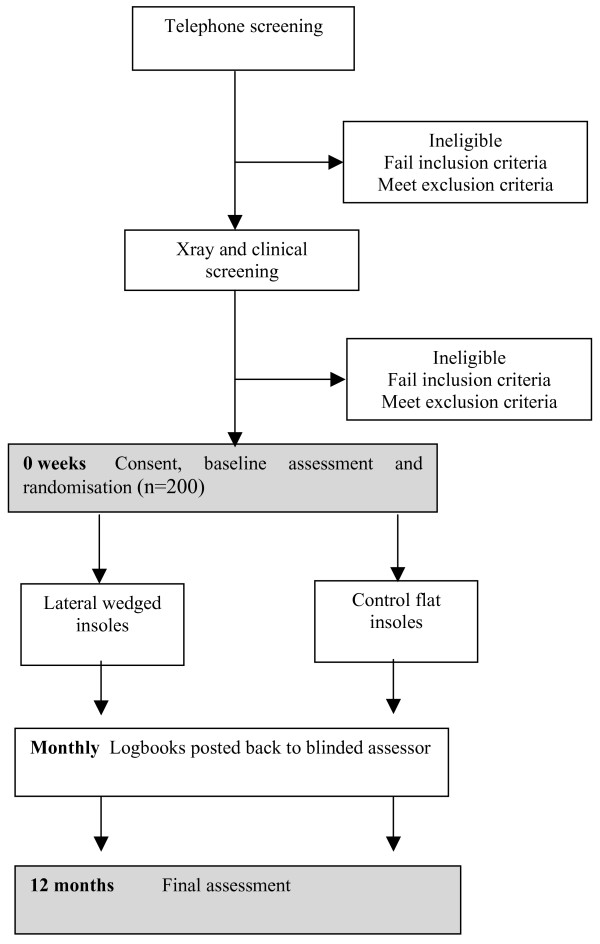
Trial protocol.

This will be a double-blind randomised controlled trial (Figure 1). Potential participants will undergo telephone screening followed by clinical examination by a physiotherapist or medical practitioner and a podiatrist to ensure they fulfil selection criteria. A screening x-ray will be performed to assess knee joint alignment and disease severity. Eligible participants will then be stratified by disease severity (Kellgren and Lawrence grade 2 and 3 [31] and randomly allocated in permuted blocks of 6 to 12 to lateral wedge or control flat insoles groups. The randomisation sequence will be generated *a priori *using the random number function in Excel by an independent investigator not directly involved in assessment of participants. Allocation will be sealed in opaque and consecutively numbered envelopes held in a central location. These will be opened in sequence by a person not involved in the study after recruitment and baseline testing of participants.

### Participants

Two hundred men and women aged over 50 years will be recruited from the community via advertisements in local clubs, libraries, and the print and radio media in metropolitan Melbourne, Australia. Eligibility will be confirmed by radiographic and clinical examination. People with tibiofemoral joint OA fulfilling American College of Rheumatology classification criteria [32] and reporting average knee pain on walking >3 on an 11-point scale will be included. Other inclusion criteria will be: (i) knee alignment ≤185° on a standardised semiflexed standing posteroanterior knee x-ray (which corresponds to a mechanical axis of ≤180° on a full leg xray indicating varus malalignment) [33]; (ii) predominance of pain/tenderness over the medial region of the knee and; (iii) medial radiographic OA defined as at least Grade 1 medial joint space narrowing or Grade 1 medial tibial or femoral osteophytes [34].

The exclusion criteria will include: (i) questionable or advanced radiographic knee OA (Kellgren and Lawrence stages 1 and 4 [31]); (ii) predominant patellofemoral joint symptoms based on clinical examination; (iii) knee surgery or intra-articular corticosteroid injection within 6 months; (iv) current or past (within 4 weeks) oral corticosteroid use; (v) systemic arthritic conditions; (vi) history of tibiofemoral/patellofemoral joint replacement or tibial osteotomy; (vii) any other muscular, joint or neurological condition affecting lower limb function; (viii) ankle/foot pathology or pain that precludes the use of insoles; (ix) use of foot orthotics within past 6 months; (x) regular use of footwear that does not accommodate an insole; (xi) contraindications to magnetic resonance imaging (eg. metal implant, cochlear implant, claustrophobia, pacemaker); (xii) planning to commence exercise or other treatment for knee OA in the next 12 months; (xiii) regular use of a gait aid and; (xiv) non-English speaking.

Ethical approval has been obtained from the University of Melbourne Human Research Ethics Committee (HREC No. 050031) and from the Department of Human Services Victoria, Radiation Safety Committee. All participants will provide written informed consent.

### Interventions

Participants will be informed that we are testing two different types of shoe insoles and will be blinded as to the types of insoles or their perceived clinical effects. To become accustomed to insole use over the first two weeks, participants will commence with one hour per day thereafter increasing by one hour per day. Participants will be asked to wear the insoles full time in their shoes. Insoles will be replaced every four months.

Participants in the intervention group will be provided with two pairs of bilateral standardised laterally wedged (5°) insoles made of high density ethyl vinyl acetate (similar to a running shoe midsole) wedged along the entire lateral border of the foot (Foot Function Ltd, New Zealand). The 5° wedge has been selected because greater wedging is unlikely to be tolerated by the wearer [24] and would be difficult to accommodate within a normal shoe.

Participants in the control group will be provided with two pairs of full length bilateral standardised footshaped flat insoles made of low density ethyl vinyl acetate (Foot Function Ltd, New Zealand). This is easily compressible soft foam that will not alter foot mechanics.

### Outcome assessment

Participants will be assessed at baseline and at 12 months by an assessor blinded to group allocation. Outcome measures have been selected based on those recommended for clinical trials of knee OA [35,36]. In those with bilateral knee OA, only the most symptomatic knee will be assessed.

Age, gender, duration of knee OA symptoms, previous treatment, surgery and medication use for knee OA will be obtained by questionnaire. Radiographic disease severity will be assessed from the baseline x-ray using the Kellgren and Lawrence grading system [31]. Skyline knee x-rays performed in nonweightbearing at 20° knee flexion will also be obtained to assess severity of OA within the patellofemoral joint. X-rays will be evaluated according to osteophytes and joint space narrowing based on a 4 point scale [34].

The primary outcomes will be knee pain and knee cartilage volume (Table 1). Average knee pain in the past week will be self-assessed by an 11-point horizontal numeric rating scale with terminal descriptors of zero = no pain and 10 = worst pain possible. Such measurement has demonstrated reliability in OA [35].

**Table 1:** Outcome measures

**Primary Outcomes**	**Measurement**
Average pain in the past week	11 point horizontal numeric rating scale (end descriptors of 0 = no pain and 10 = worst pain possible)
Medial tibial compartment cartilage volume	Magnetic resonance imaging

**Secondary Outcomes**	

Pain, stiffness and physical function in past 48 hours	WOMAC Osteoarthritis Index – Likert version
Global perceived response to treatment for pain and for function	Ordinal scale (1-much worse, 2-slightly worse, 3-no change, 4-slightly better, 5-much better) at study completion
Health-related quality of life	Assessment of Quality of Life index (AQol)
Physical activity levels	• Physical Activity Scale for the Elderly (PASE) • Pedometer worn twice for one week

**Other measures**	

Compliance	• Daily hours of use recorded in log-book • 11 point horizontal numeric rating scale (end descriptors of 0 = not at all and 10 = completely as instructed) at study completion
Discomfort with insoles	• Assessed weekly in log-book on 5-point ordinal scale (1 = no discomfort, 2 = mild, 3 = moderate, 4 = severe, 5 = very severe discomfort) • 11 point horizontal rating scale (end descriptors of 0 = extremely comfortable and 10 = extremely uncomfortable) at study completion
Other adverse effects	Log-book and open probe questionning

Knee cartilage volume will be measured using magnetic resonance imaging (MRI). Knees will be imaged in a sagittal plane on a 1.5-T whole-body magnetic resonance unit (Signa Advantage HiSpeed GEMedical Systems, Milwaukee, WI, USA) using a commercial receive-only extremity coil. The following sequence and parameters will be used: a T_1_-weighted, fat-suppressed 3D gradient recall acquisition in the steady state; flip angle 55 degrees; repetition time 58 ms; echo time 12 ms; field of view 16 cm; 60 partitions; 512 (frequency direction, superior-inferior) × 512 (phase encoding direction, anterior-posterior) matrix; one acquisition, time 11 min 56 sec. Sagittal images will be obtained at a partition thickness of 1.5 mm and an in-plane resolution of 0.31 mm × 0.31 mm (512 × 512 pixels) [37]. Knee cartilage volume will be determined by a blinded assessor analyzing baseline and final MRI pairs. 3D image processing will be performed using the software program OSIRIS (University of Geneva). In this technique, the image data will be transferred to a workstation and an isotropic voxel size will then be obtained by a trilinear interpolation routine. The volume of individual cartilage plates will be isolated from the total volume by manually drawing disarticulation contours around the cartilage boundaries on a section-by-section basis. These data will then be resampled by means of bilinear and cubic interpolation (area of 312 × 312 μm and 1.5 mm thickness, continuous sections) for the final 3D rendering. The volume of the particular cartilage plate will then be determined by summing all the pertinent voxels within the resultant binary volume. The CV of this method in our hands is 2% [37]. Knee cartilage volume will be measured for both the medial and lateral tibial compartments but the primary outcome will be the change in medial tibial cartilage volume. Bone size is a significant determinant of knee cartilage volume and thus a potential confounder [38]. Medial and lateral tibial plateaux areas as a measure of bone size will determined by creating an isotropic volume from the sagittal, T1-weighted fat saturation images, described above. This data will be reformatted in the axial plane and the area directly measured from these images as previously described [19]. The CV for the tibial plateau areas is 2.3% [19].

A number of secondary outcomes will be used (Table 1). The Western Ontario and McMaster Universities (WOMAC) Osteoarthritis Index is a disease-specific instrument widely used in clinical trials, outcomes research and epidemiological surveys. Its validity, reliability and responsiveness have been demonstrated in an extensive range of studies [39]. The WOMAC consists of 24 questions covering pain, stiffness and physical function. It provides a total score as well as scores for each subscale.

The Assessment of Quality of Life (AQoL) instrument is designed to measure health-related quality of life. It has 15 questions that cover five dimensions including illness, independent living, social relationships, physical senses and psychological wellbeing. The AQoL has strong psychometric properties and is more responsive than other widely-used scales including the Medical Outcomes Study 36-Item Short Form (SF-36) [40,41]. It produces a single utility index that ranges from -0.04 (worst possible health-related quality of life) to 1.00 (full health-related quality of life). A clinically important difference in health related quality of life can be defined as a change of 0.04 AQoL units [42]. Population norms have been calculated for the Australian population [42].

Habitual physical activity will be measured in two ways, one using a questionnaire and the second using a pedometer. The Physical Activity Scale for the Elderly (PASE) will be used to measure both the level and type of recreational and occupational physical activity undertaken by participants over the previous week. The PASE was developed and validated in samples of older adults (age 55+ years) [43]. A pedometer (HJ-005 Omron Healthcare, Japan) will be worn at the waist for a week on two occasions, once at the beginning and once at the end of the 12-month study to record the number of steps taken per day. Participants will be asked to wear the pedometer full time during their waking hours. Pedometers have been found to be a simple and inexpensive means to estimate physical activity levels [44,45]. It is recommended that at least three days of sampling are needed to accurately assess activity levels given differences between weekends and weekdays [46].

Participants will rate their perceived change in pain and in physical function with treatment (compared to baseline) on an ordinal scale (1-much worse, 2-slightly worse, 3-no change, 4-slightly better, 5-much better). Scales of this kind are frequently used as an external criterion for comparison with changes in scores of other outcomes [47]. Measuring patient perceived improvement using a rating of change scale has been shown to be a clinically relevant and stable concept for interpreting truly meaningful improvements from the individual perspective [48].

A number of other measures will be obtained (Table 1). Participants will record compliance of and tolerance with both types of insoles in a daily log-book. They will record the number of hours per day of insole use. At the end of the 12 months, participants will also indicate their perceived compliance with the insoles using an 11 point horizontal rating scale with terminal descriptors of zero = not at all and 10 = completely as instructed. Discomfort with the insoles will be evaluated weekly in the logbook on a 5-point ordinal scale (1 = no discomfort, 2 = mild, 3 = moderate, 4 = severe, 5 = very severe discomfort) [28]. At the end of the 12 months, participants will also rate their overall level of discomfort of the insoles using an 11 point horizontal rating scale with terminal descriptors of zero = extremely comfortable and 10 = extremely uncomfortable. Other adverse effects and the use of co-intervention will be recorded in the log-book and by open-probe questioning by the assessor at trial completion. Log-books will be posted back to the assessor on a monthly basis and checked for completion.

### Sample size calculations

Our sample size of 200 ensures adequate power for the two principal endpoints of change in pain and change in knee cartilage volume. Assuming that lateral wedges result in a mean pain reduction of 2 on our numeric rating scale (the minimum clinically important difference to be detected in OA trials [49]), that control insoles have a small placebo effect of 0.5 and that there is a SD of change of 3 (based on our previous data evaluating conservative OA interventions [50,51]), 170 participants would be required in total to give 80% power (2 sided test, alpha = 0.05). We have previously demonstrated a reduction in pain of this magnitude with knee taping [50] and physiotherapy for knee OA [51].

The other major end point is rate of knee cartilage loss. We have previously shown that the rate of tibial cartilage loss is 5.3 +/- 5.2% per year in a cohort with knee OA [52]. We categorized OA participants into tertiles: cartilage loss <3% per annum, 3–8% per annum and > 8% per annum. We found a linear increase in the risk of undergoing a knee replacement within 4 years [53]. Participants who lost cartilage at the rate of 3–8% per annum had a 2.4 fold (0.4 to 12.2) increased risk of requiring a knee replacement compared to participants who lost cartilage at the rate of <3% per annum. Those with the highest rate of loss of knee cartilage (>8% per annum) had a 7.1 fold (1.4 to 36.5) increased risk of requiring a knee replacement. These data suggest that a clinically useful outcome with lateral wedges would be to reduce the rate of knee cartilage loss to <3% per annum. With 100 subjects in each arm of the study, we will have 80% power to show a difference between a 5.3 +/- 5.2% per year rate of cartilage loss in the control group [52] and a <3% rate of cartilage loss in the intervention group (2 sided test, alpha = 0.05). This also takes into account a 20% drop-out rate.

### Economic analysis

The economic analysis will be conducted from the perspective of the Australian health care system and the individual patient. Participants will record visits to health care providers (e.g. general practitioner, medical specialist, other health care professionals), prescription and over the counter medication, professional home care and hospitalization in a daily log-book. This will be completed and returned to the assessor on a monthly basis. The log-book will utilise a checklist format to minimize respondent burden and ensure high quality data with minimal data loss.

### Statistical analysis

A blinded statistician will analyse the data. All analyses will be conducted on an intention-to-treat principle using all randomized participants. Missing data will be replaced by the last score carried forward. Demographic characteristics and baseline data will be summarised by descriptive statistics. For outcomes measured using an essentially continuous scale, differences in mean change from baseline to 12 months will be compared between groups using linear regression modelling adjusting for baseline levels of the outcome measure. For knee cartilage volume, bone area, age and gender will also be included as covariates [54,55]. Model assumptions will be checked by standard diagnostic plots [56]. Participant measures of perceived improvement following lateral wedges or flat insole treatments will be compared by calculating the relative risks and their 95% confidence intervals using log binomial regression [57].

The primary economic evaluation will take the form of a cost effectiveness study with a range of outcome measures including the incremental cost per extra person with a clinically significant improvement in pain, per extra person perceived to be recovered, and per extra quality adjusted life years (using the AQoL over 12 months). A social perspective on costs will be taken that includes resource use incurred both by health services and by the participant irrespective of the source of payment. The inclusion of time/productivity gains is controversial and the cost effectiveness ratios will be calculated with and without these indirect costs. All health care costs will be included, however, to reduce the impact of extreme values, if inpatient hospital costs are unrelated to knee OA they will be excluded. Standard methods of economic evaluation alongside a clinical trial [58] will be used to evaluate the differences in resource use and health outcomes over 12 months between groups. The statistical analysis of costs data will be similar to outcome data although adjustments for overdispersion may be necessary. Confidence intervals for incremental cost effectiveness will be calculated directly using non-parametric bootstrapping [59]. In addition we will calculate a cost effectiveness acceptability curve based for a range of hypothetical money values of outcomes [60]. This will be done using individual cost and outcome data over the 12 months or, if adjustments for imbalance at baseline are necessary, using regression analysis [61]. Hypothetical money values will be taken from the decision making literature but the trial will also ask patients in each arm of the trial their willingness to pay for the treatment prior to and after treatment. This will not only provide money values for the calculation of net benefits but also provide evidence on the influence of health experience on the value of health outcome to patients.

## Discussion

This study uses a double-blind randomised controlled trial design to investigate whether lateral wedge insoles have greater effects on symptoms and disease progression and are more cost-effective than control flat insoles in people with mild to moderately severe medial knee OA and varus malalignment.

Recent research has highlighted the existence of subgroups of patients with knee OA particularly with regards to local factors such as malalignment. It is known that these subgroups show different risks of progression and may respond differently to interventions [62]. Thus, our inclusion/exclusion criteria aim to recruit a subgroup of people with medial knee OA that would most likely respond to lateral wedge insoles. We excluded those with severe disease because there is little scope for structural progression, one of our primary outcomes and recent studies have also suggested that benefits with wedges might be confined to those with mild to moderate disease [63].

Lateral wedge insoles may vary in their angle of wedge and in their length. Whilst a higher lateral wedge may produce greater biomechanical effects, it is difficult to fit comfortably inside the shoe. We are testing a full length wedge rather than a heel wedge because we found that in 13 people with medial knee OA, a full length wedge reduced the knee adduction moment by 12% compared to no insoles whereas moments obtained with rearfoot wedges were not significantly different to those obtained without insoles (Hinman et al unpublished data).

Our choice of outcome measures are those recommended for use in clinical trials of OA by international rheumatology bodies [35,36]. These include measures of pain, function, quality of life and global response to treatment. Magnetic resonance imaging represents an advance in the measurement of structural disease progression. Traditionally, serial x-rays have been used to measure loss of joint space over time. However, measuring joint space width using conventional radiography provides only an approximation of articular cartilage, while MRI directly visualizes joint cartilage. MRI is recognised as a valid, accurate and reproducible tool to measure articular cartilage volume [38,64]. It is sensitive to change in both normal subjects [65] and those with OA [52] and has been shown to relate to clinically relevant end points including worsening of knee symptoms [66] and the risk of knee replacement [53].

It is anticipated that all participants will be recruited by the end of 2007 with data acquisition completed a year later. The results from this trial will contribute to evidence based recommendations for the usefulness of lateral wedged insoles in the management of medial knee OA.

## Competing interests

The author(s) declare that they have no competing interests.

## Authors' contributions

KB and RH conceived and designed the trial protocol. KB, RH, FC, CP and AH procured the project funding. CP was responsible for the podiatric screening assessment and the insole design and acquisition. FC designed the MRI data acquisition and analysis. AH designed the economic analysis. RO designed the statistical analysis. K-AB is the research assistant on the project. KB drafted the manuscript and RH, CP, FC, K-AB, AH and RO contributed to the manuscript. All authors read and approved the final manuscript.

## Pre-publication history

The pre-publication history for this paper can be accessed here:


